# The challenge of extraabdominal desmoid tumour management in patients with Gardner’s syndrome: radiofrequency ablation, a promising option

**DOI:** 10.1186/1477-7819-12-361

**Published:** 2014-11-27

**Authors:** Lorenzo Cobianchi, Valentina Ravetta, Francesca Torello Viera, Claudia Filisetti, Barbara Siri, Edoardo Segalini, Marcello Maestri, Tommaso Dominioni, Mario Alessiani, Sandro Rossi, Paolo Dionigi

**Affiliations:** General Surgery 1, Fondazione IRCCS Policlinico San Matteo, Viale Camillo Golgi 19, 27100 Pavia, Italy; VI Department of Internal Medicine, Fondazione IRCCS Policlinico San Matteo, Pavia, Italy; Paediatric Surgery Department, Children’s Hospital Vittore Buzzi, Milan, Italy; School of Medicine, University of Pavia, Pavia, Italy

**Keywords:** Desmoid tumours, Gardner’s syndrome, Radiofrequency ablation

## Abstract

Desmoid tumours are benign, myofibroblastic stromal neoplasms common in Gardner’s syndrome, which is a subtype of familial adenomatous polyposis characterized by colonic polyps, osteomas, thyroid cancer, epidermoid cysts, fibromas and sebaceous cysts. The primary treatment is surgery, followed by adjuvant radiotherapy, but the local recurrence rate is high, and wide resection can result in debilitating loss of function. We report the case of a 39-year-old man with Gardner’s syndrome who had already undergone a total prophylactic colectomy. He developed desmoid tumours localized in the mesenteric root, abdominal wall and dorsal region, which were treated from 2003 through 2013 with several surgical procedures and percutaneous radiofrequency ablation. In 2008 and 2013, RFA was applied under ultrasonographic guidance to two desmoid tumours localized in the dorsal thoracic wall. The outcomes were low-grade pain and one case of superficial skin necrosis, but so far there has been no recurrence of desmoid tumours in these locations. Surgical resection remains the first-line therapy for patients with desmoid tumours, but wide resection may lead to a poor quality of life. Radiofrequency ablation is less invasive and expensive and is a possible therapeutic option for desmoid tumours in patients with Gardner’s syndrome.

## Background

Desmoid tumours (DTs) are uncommon, histologically benign, myofibroblastic neoplasms that arise from musculoaponeurotic stromal elements. DTs occur rarely in the general population, accounting for approximately 0.03% of all neoplasms and less than 3% of all soft-tissue tumours. The estimated incidence of the spontaneous form is 2million to 4 per million per year [1–3], but DTs are relatively common in patients with familial adenomatous polyposis (FAP), with an incidence of 3.5% to 32% and a higher incidence of 29% in the original kindred with Gardner’s syndrome [4]. Gardner’s syndrome (GS) is a FAP subtype characterized by a high occurrence of DTs, colonic polyps and extraabdominal tumours, including osteomas of the skull, thyroid cancer, epidermoid cysts, fibromas and sebaceous cysts [5]. DTs can develop anywhere in the body and generally occur in intra- and extraabdominal anatomical locations. The most common locations are the extremities (around the limb girdles and proximal extremities), the abdominal wall and intraabdominal and mesenteric sites. Depending on the location, DTs tend to infiltrate adjacent organs, extend along fascial planes and compress blood vessels and nerves, creating severe symptoms such as intestinal obstruction and bowel ischemia [6–8]. The biological behaviour of DTs, such as growth and recurrence rates and age and sex predilection, are considered unpredictable and vary primarily by location. Local recurrence rates for intraabdominal tumours are higher than those of extraabdominal tumours, reported to be 57% to 86% [9, 10]. Despite such extant data, the natural history of DTs remains poorly understood [11]. The first-line therapy for patients with locally circumscribed DTs remains surgical resection. The standard surgical goal is complete resection with negative microscopic margins; however, wide resection can result in debilitating loss of function. The boundaries of the tumours are difficult to distinguish intraoperatively from scars and connective tissue, so R0 resection is not always possible, and, consequently, adjuvant radiotherapy is often applied. DTs, however, have a high local recurrence rate after surgery and/or radiotherapy. In this report, we introduce radiofrequency ablation (RFA) as a treatment option for DTs in a patient with GS.

## Case presentation

**Figure 1:**
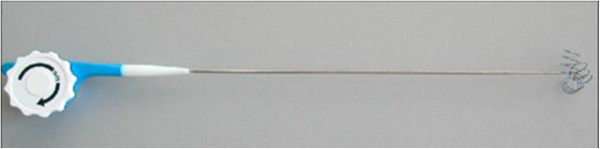
**Flexible radiofrequency ablation 2.8-mm cooled-wet electrode used for radiofrequency ablation of the desmoid tumours.**

**Figure 2:**
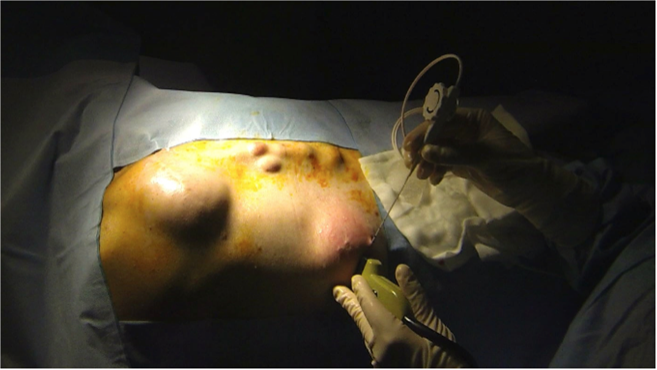
**Intraoperative photography taken during the radiofrequency ablation procedure performed with ultrasonographic guidance.**

**Figure 3:**
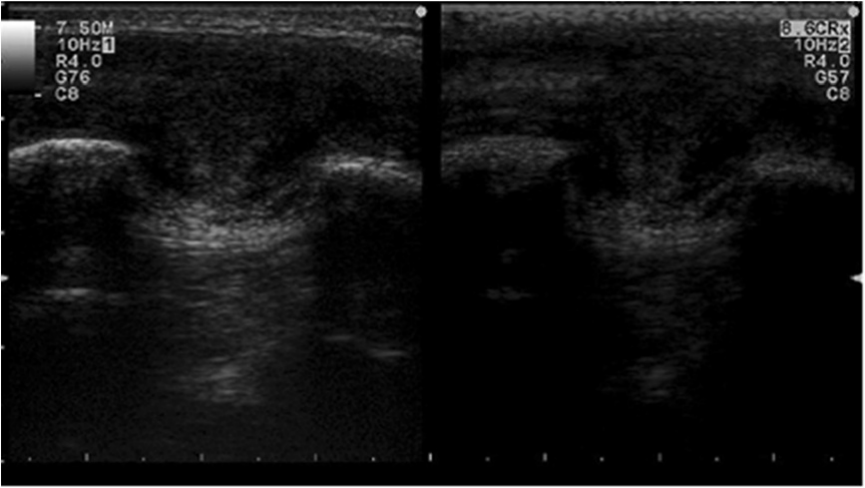
**Postoperative contrast-enhanced ultrasonographic image.** Double image that shows the results after radiofrequency ablation of the desmoid tumour under contrast-enhanced ultrasonographic guidance (SonoVue; Bracco Imaging, Milan, Italy). The absence of contrast enhancement demonstrates the successful treatment.

A 39-year-old man with GS was referred to our institution in April 2002. He was known to have a positive family history of GS. In 1996, he underwent a total prophylactic colectomy and subsequently developed DTs localized in the mesenteric root, abdominal wall and dorsal region. These DTs were judged to be untreatable by another medical centre and were treated with sulindac in our oncologic department without any benefit. In 2002, the patient was referred to our centre because of the presence of multiple giant DTs in the abdominal wall, which had caused abdominal visceral compression and intestinal obstruction. The patient underwent a successful radical surgical resection of the abdominal DTs. Since 2005, the patient had experienced recurrence of DTs in the previous location and in the right lateral thoracic wall and right infrascapular and left subscapular regions. We decided on two treatment approaches for these masses: surgical removal of the entire tumours and percutaneous RFA. Between 2003 and 2013, several surgical interventions for desmoid mass excision were carried out. In 2008 and 2013, RFA was planned and applied to two DTs localized in the dorsal thoracic wall. The systems used in the RFA procedures have been described in detail elsewhere [12, 13]. In brief, they utilize a commercially available RF generator (Model TAG 100, Invatec Srl, Roncadelle, Italy, or Model RF 3000, Boston Scientific, Natick, MA, USA), a grounding pad (conductive plates measuring 8.0 × 16.5 cm; GPS Srl, Mozzo, Italy) and active electrodes. In the 2008 treatment session, we performed the procedure using a flexible, RFA, 2.8-mm, cooled-wet electrode. The active tip is a stiff, straight, hollow 2.8-mm, stainless steel cannula which contains a single monopolar electrode and a perfusion system in a single device (Figure 1). In February 2013, we used an expandable needle electrode (Miras TX model, 120 × 25 mm, 17-gauge), with stainless steel shafts (15 to 25 cm long) insulated with a 0.1-mm-thick layer of plastic and exposed tips (1.0 mm long). The shafts contain three nickel titanium spiral extensions which can be fully or partially deployed and retracted using a lever on the electrode handle. The RFA technique that we performed was the same as described elsewhere for the ablation of liver tumours [12]. The procedure was performed with the patient under general anaesthesia, induced following best clinical practice norms. When using the flexible RFA cooled-wet electrode, the tip was inserted under ultrasonographic guidance into the distal part of the tumour along the lesion’s major axis and retracted using a pull-back technique. The generator was then activated to deliver 70 to 100 W for 3 to 6 minutes. When the 17-gauge, triple-spiral electrode was used, the tip was inserted perpendicular to the lesion, advanced until the tip was seen in the centre of the tumour, and deployed. The radiofrequency generator was then activated to deliver 20 to 50 W for 6 to 12 minutes. To ensure ablation of the full circumference and to avoid causing burns, we cooled the skin over the tumour with ice throughout the procedure (Figure 2). When the hyperechoic zone around the electrode tip was as large as the tumour, the procedure was terminated and the electrode was withdrawn. The patient’s pain was self-limiting, and the only complication was a small first-degree burn at the point of insertion of the electrode needle. Since this procedure, there has been no recurrence of DTs after 18 months of follow-up at our centre (Figure 3). The patient is in good clinical condition and receiving routine follow-up. For the imaging modality during the follow-up, we used ultrasonography and contrast-enhanced ultrasonography.

## Conclusions

In patients with GS, the treatment of DTs is an unresolved problem. Even if DTs are histologically benign in GS, they have real clinical malignancy. Many therapies have been proposed—surgical resection, pharmacotherapy and radiation treatment—but none have produced satisfactory outcomes. The first-line therapy for patients with locally circumscribed DTs remains surgical resection. The standard surgical goal is complete resection with negative microscopic margins, but wide resection can result in debilitating loss of function. The boundaries of the tumours are difficult to distinguish intraoperatively from scars and connective tissue, so R0 resection is not always possible, and, consequently, adjuvant radiotherapy is often applied [14]. In particular, surgery has been related to an increased incidence of recurrence, and some authors have demonstrated the role of surgical trauma in DT formation in GS [15, 16]. To avoid mutilating surgical interventions and the potential negative effects of surgical trauma, various pharmacotherapeutic approaches have been proposed—hormonal therapy, nonsteroidal anti-inflammatory drugs (for example, celecoxib and sulindac), cytotoxic chemotherapy and tyrosine kinase inhibitors, such as imatinib and sorafenib—but none have produced successful results [17, 18]. In all therapies, the primary aim is to preserve the patient’s quality of life. To date, treatment of DTs remains controversial, and an optimal therapeutic protocol for this disease has not been established. Because of the rarity DTs and the practical limitations in conducting research on them, they often evade accurate characterization. On the basis of the previous considerations, we attempted two minimally invasive treatments for our patient—percutaneous cryoablation and RFA. Percutaneous cryoablation has been shown to be an effective alternative treatment for the local control of small and moderately sized extraabdominal DTs, although it is contraindicated in patients with large tumours involving vital structures [19]. The ablation treatment of a series of patients with paraspinal and limb DTs has recently been reported. RFA was reported to have produced effective local control with no recurrence and only one complication of superficial skin necrosis in 30 months of follow-up [15, 16, 20–23]. In another recent study, the authors reported the use of this technique to debulk and palliate an abdominal wall DT in a patient with FAP [24]. Compared with surgery, RFA is less invasive and less expensive, requires a shorter anaesthesia time and does not require use of an operating room. We decided to introduce RFA as a treatment option to destroy tissue without stimulating neuromuscular reaction. On the basis of our experience and the recent literature, we propose that RF thermoablation stands as a possible therapeutic option in the highly challenging treatment of DTs in patients with GS. We need to perform more of these procedures to define clearly the role of this approach in the treatment of patients with DTs.

## Consent

Written informed consent was obtained from the patient for publication of this case report and any accompanying images. A copy of the written consent is available for review by the Editor-in-Chief of this journal.

## Abbreviations

DT: Desmoid tumour
FAP: Familial adenomatous polyposis
GS: Gardner’s syndrome
RFA: Radiofrequency ablation
US: Ultrasonography.
